# Effective therapeutic targeting of *CTNNB1*‐mutant hepatoblastoma with WNTinib

**DOI:** 10.1002/1878-0261.70168

**Published:** 2025-12-08

**Authors:** Ugne Balaseviciute, Júlia Huguet‐Pradell, Jordi Abril‐Fornaguera, Albert Gris‐Oliver, Alex Rialdi, Elisa Fernández‐Martínez, Carla Montironi, Vanessa Del Pozo, Peter Houghton, Laura Zanatto, Agavni Mesropian, Ieva Keraite, Swan Thung, Carolina Armengol, Pau Sancho‐Bru, Ernesto Guccione, Roser Pinyol, Josep M. Llovet

**Affiliations:** ^1^ Liver Cancer Translational Research Group, Institut d'Investigacions Biomèdiques August Pi i Sunyer (IDIBAPS), Hospital Clínic Universitat de Barcelona Catalonia Spain; ^2^ Facultat de Medicina i Ciències de la Salut Universitat de Barcelona Catalonia Spain; ^3^ Mount Sinai Liver Cancer Program (Divisions of Liver Diseases, Department of Hematology/Oncology, Department of Medicine, Department of Pathology) Tisch Cancer Institute, Icahn School of Medicine at Mount Sinai New York NY USA; ^4^ Pathology Department and Molecular Biology Core Hospital Clínic of Barcelona Spain; ^5^ Greehey Children's Cancer Research Institute University of Texas Health Science Center San Antonio TX USA; ^6^ Institut d'Investigacions Biomèdiques August Pi i Sunyer (IDIBAPS) Universitat de Barcelona Spain; ^7^ Department of Pathology ISMMS New York NY USA; ^8^ Childhood Liver Oncology Group (c‐LOG) Health Sciences Research Institute Germans Trias i Pujol (IGTP) Badalona Spain; ^9^ Liver and Digestive Diseases Networking Biomedical Research Centre (CIBEREHD) Madrid Spain; ^10^ Center for Therapeutics Discovery, Department of Oncological Sciences and Pharmacological Sciences Tisch Cancer Institute, Icahn School of Medicine at Mount Sinai New York NY USA; ^11^ Center for OncoGenomics and Innovative Therapeutics Tisch Cancer Institute, Icahn School of Medicine at Mount Sinai New York NY USA; ^12^ The Precision Immunology Institute Icahn School of Medicine at Mount Sinai New York NY USA; ^13^ Bioinformatics for Next Generation Sequencing Shared Resource Facility Icahn School of Medicine at Mount Sinai New York NY USA; ^14^ Institució Catalana de Recerca i Estudis Avançats Barcelona Catalonia Spain

**Keywords:** β‐Catenin (CTNNB1)‐mutated, hepatoblastoma (HB), multi‐kinase Wnt inhibitor, targeted treatment, WNTinib

## Abstract

Hepatoblastoma (HB), the most frequent pediatric liver cancer (2.16 cases/million), has surgery and perioperative chemotherapy as primary treatment, with severe lifelong side effects. This study evaluates the efficacy of the Wnt/*CTNNB1* inhibitor WNTinib as a potential HB treatment, since *CTNNB1* mutations occur in 70–90% of HBs. WNTinib's efficacy was assessed in three animal models (*n* = 48): (a) patient‐derived xenograft (PDX) HB tumors (*n* = 5 *CTNNB1‐*mutant, *n* = 1 *CTNNB1* wild‐type) implanted in NSG mice; (b) PDX‐derived TT001‐ and (c) HepG2‐HB cells subcutaneously implanted in Fox1^nu^ mice; and in two patient‐derived organoids from *CTNNB1*‐mutant HBs. WNTinib delayed tumor growth in *n* = 4/5 *CTNNB1*‐mutant PDX models and significantly improved survival versus controls (*P* = 0.03), with no effect in the wild‐type model. Further, in the TT001 and HepG2 models, WNTinib reduced tumor growth (*P* < 0.05 and *P* = 0.002) and extended survival (*P* = 0.03 and *P* = 0.008), respectively. In HB organoids, WNTinib demonstrated greater efficacy than standard‐of‐care cisplatin (*P* = 0.009, org‐1), and its antitumor effect was further enhanced when combined with chemotherapy (*P* = 0.01, org‐1; *P* = 0.007, org‐22). WNTinib delays tumor progression and increases survival in *CTNNB1*‐mutated HB models, providing rationale to explore its use in human HB.

## Graphical abstract

WNTinib, a Wnt/CTNNB1 inhibitor, was tested in hepatoblastoma (HB) experimental models. It delayed tumor growth and improved survival in *CTNNB1*‐mutant *in vivo* models. In organoids, WNTinib outperformed cisplatin and showed enhanced efficacy in combination therapy, supporting its potential as a targeted treatment for *CTNNB1*‐mutated HB.

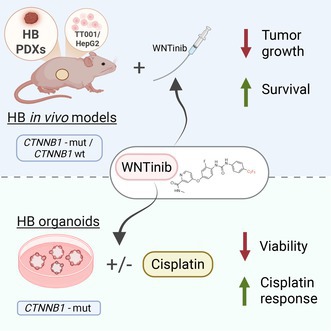

**Abbreviations**
*APC*
: Adenomatous Polyposis Coli
BME: Basement Membrane Extract
CR: complete responder
*CTNNB1*
: catenin beta 1
EFS: event‐free survival
EZH2: Enhancer of Zeste Homolog 2
FDR: False Discovery Rate
FFPE: formalin‐fixed paraffin‐embedded
HB: Hepatoblastoma
HCC: hepatocellular carcinoma
IGF2: insulin‐like growth factor 2
mRECIST: Modified Response Evaluation Criteria in Solid Tumors
*NFE2L2*
: nuclear factor erythroid 2‐related factor 2
OR: objective response
PDO: patient‐derived organoid
PDX: patient‐derived xenograft
SCID: Severe Combined Immunodeficiency
*TERT*
: Telomerase Reverse Transcriptase
WT: wild type

## 1 Introduction

Hepatoblastoma (HB) is the most common primary liver cancer in children, albeit rare, with an incidence of 1.5–2.16 cases per million children per year [1, 2]. The standard treatment includes liver surgery combined with neoadjuvant and/or adjuvant platin‐based chemotherapy (e.g., cisplatin or cisplatin plus doxorubicin) [3]. The overall survival of HB patients varies across HB stages at diagnosis. Patients at more advanced stages, who are ineligible for liver surgery, have a 5‐year event‐free survival (EFS) rate of 35–40% [1, 4]. Additionally, survivors often face severe lifelong secondary effects derived from chemotherapy, such as ototoxicity and cardiomyopathy [5, 6], which have impactful consequences on the quality of life of HB patients. Genomically, HB is one of the neoplasms with the lowest rates of somatic mutation (2.9 mutations/tumor) [7, 8], with mutations affecting the WNT/β‐catenin pathway as the most prevalent ones (*CTNNB1* mutations present in 70–90% of HB tumors; *AXIN1* and *APC*, present in <5%) [9, 10]. Other frequent alterations include changes in the 11p15.5 locus, causing IGF2 overexpression (70%) [11, 12]; or mutations in driver genes such as *NFE2L2* (9.8%) and *TERT* (6%) [9, 10].

Mutations in *CTNNB1*, the gene encoding for β‐catenin, have been shown to activate the WNT/β‐catenin pathway and promote the transcription of genes involved in metabolism and tumor proliferation [13, 14]. The high frequency of *CTNNB1* mutations in HB suggests that patients could benefit significantly from therapies targeting the WNT/β‐catenin pathway [15, 16]. However, targeting this pathway remains challenging due to its complexity (involving 19 ligands and 15 receptors) [17] and its role in several critical biological functions [14, 17]. Currently, no WNT/β‐catenin inhibitors have been approved for clinical use.

Recently, we identified a WNT/β‐catenin inhibitor, WNTinib, which presents high selective antitumoral efficacy and a favorable safety profile in *CTNNB1*‐mutated preclinical models of hepatocellular carcinoma (HCC) [18]. WNTinib is a multi‐kinase inhibitor derived from sorafenib and regorafenib [19, 20, 21], two clinically approved drugs for HCC. In HCC, WNTinib targets kinases such as c‐kit, blocking the phosphorylation of the transcriptional repressor EZH2. Subsequently, the dephosphorylated EZH2 translocates to the nucleus and inhibits the transcription of WNT/β‐catenin target genes [18]. Given the high prevalence of *CTNNB1* mutations in HB and the observed effect of WNTinib in HCC, we aimed to evaluate the antitumoral effect of WNTinib in HB preclinical models. The results of our study show that WNTinib effectively delays tumor progression and increases survival in three independent *in vivo* models of *CTNNB1*‐mutated HB and shows efficacy in patient‐derived HB *CTNNB1*‐mutated organoids.

## 2 Materials and methods

### 2.1 Patient‐derived xenograft (PDX) murine models

Immunodeficient patient‐derived xenograft (PDX) murine models of HB were generated by subcutaneously engrafting six patient‐derived HB xenografts into 6–8‐week‐old female C.B‐Igh‐1b/IcrTac‐Prkdcscid (SCID; RRID:IMSR_TAC:CB17SC) mice obtained from Jackson Laboratory (Maine, USA). The study was undertaken with the understanding and written consent of the parent or legal guardian of each patient, as all tumor samples were obtained from pediatric subjects aged 5 to 45 months. Samples were collected between March 2017 and July 2022 from the University Hospital, San Antonio, TX, and the University of Texas Southwestern, Dallas, TX (IRB Protocol Number: HSC20080057H; IACUC Protocol Number: 20150015AR). Tumor samples of 2–5 mm^3^ were implanted subcutaneously into the right flank of 12 mice. When tumors reached 200–250 mm^3^, animals were randomly assigned to receive WNTinib (*n* = 6) or control (1:1 solution of Kolliphor EL : ethanol (Sigma‐Aldrich, Steinheim, Germany) and diluted with 4 parts water) (*n* = 6). WNTinib (30 mg/kg) or vehicle were administered daily by oral gavage for 28 days. Tumor growth was monitored weekly using a caliper and tumor volumes were calculated as follows: volume = length × width^2^ × 0.5. Animal weight was monitored weekly. Animals were sacrificed at the survival endpoint, which was defined as either a tumor volume > 1000 mm^3^, the appearance of clinical symptoms, and/or severe body weight loss (10%/day). Mice treated with WNTinib experienced nonsignificant weight loss during the treatment and regained weight immediately after treatment stopped (Fig. S2A). This motivated a reduction in the WNTinib dose from 30 to 20 mg/kg in subsequent experiments.

### 2.2 Establishment of PDX‐derived 2D cell lines

Firstly, a patient‐derived HB tumor (Xentech) [22] was implanted subcutaneously in 6–8‐week‐old female NOD.CB17‐Prkdcscid/NcrCrl immunosuppressed mice (RRID:IMSR_CRL:394; Charles River Laboratories, Wilmington, MA, USA). Animals were weighed, and tumor volume was assessed three times/week using bilateral caliper measurements and the formula: volume = length × width^2^ × 0.5. Once tumors reached 1000 mm^3^, the animals were sacrificed, and tumors were collected and minced to generate the PDX‐derived cell lines. In brief, minced tumors were digested in sterile digestion media (1 mg/mL of collagenase IV in PBS) for 60 min at 37 °C. Tumor dissociates were strained through a 70‐μm strainer, washed with complete RPMI (20% FBS, 1% glutamine and 1% penicillin–streptomycin) and cells were counted. A total of 10 × 10^6^ cells were plated on collagen‐coated 10‐cm dishes in primary media (5 μm A83‐01, 10 μm Y‐27632 and 40 ng/mL of recombinant human EGF in complete RPMI). Histological and molecular characterization of the PDX from which TT001 was established has been reported elsewhere (PDX ID: HB‐235) [22].

### 2.3 Subcutaneous xenograft murine models

To generate the xenograft murine model of *CTNNB1*‐mutated HB cells, 5 × 10^6^ HepG2 cells (ATCC, Manassas, VA, USA) or 5 × 10^6^ TT001‐PDX‐derived cells suspended in 50% v/v Matrigel were subcutaneously injected into the right flank of immunocompromised 6–8‐week‐old athymic (NMRI‐Foxn1nu; RRID:MGI:5653040) female mice (*n* = 48; Charles River Laboratories, Wilmington, MA, USA). Animals were weighed weekly, and tumor volumes were measured every 2–3 days as described above. When tumors reached 200–250 mm^3^, animals were randomly assigned to receive WNTinib or vehicle. WNTinib (20 mg/kg, 200 μL) and vehicle (200 μL) were administered by oral gavage on a 5‐day‐on and 2‐day‐off treatment schedule [18]. WNTinib was prepared as reported previously [18]. Body weight, tumor growth, and survival were monitored in all mice. Once animals reached the survival endpoint (defined as 1000 mm^3^, or more than twice the duration of tumor latency), they were sacrificed.

### 2.4 Histological and immunohistochemical analyses of murine HB samples

According to standard protocols, histological analysis of HB murine tumor tissues was performed on formalin‐fixed paraffin‐embedded (FFPE) tissue sections stained with hematoxylin and eosin. Immunohistochemistry for Ki67 was performed on 5‐μm sections of FFPE blocks. Antigen retrieval was performed in a high pH buffer. After antigen retrieval, samples were incubated with peroxidase, blocked with Antibody Diluent containing Background Reducing Component (Dako), and washed with PBS‐1% Triton. Samples were incubated overnight with the anti‐Ki67 antibody from Abcam (ab16667) (1:50). EnVisionTM+ System‐HRP (DAB) was applied as a secondary antibody (Dako). Samples were counterstained with hematoxylin. Experienced pathologists (S.T. and C.M. co‐authors of the study) performed the histological evaluation of these tumors. For quantification of the Ki67, slides were scanned at 20X magnification (Hamamatsu S210 Scanner, Hamamatsu City, Japan), and digital image analysis was performed with the open‐source software QuPath v0.4.3. Viable tumor areas were selected, and the percentage of positive staining was measured.

### 2.5 Patient‐derived organoid (PDO) generation and drug viability assay

All samples from patients with HB were collected in accordance with European and Spanish law and institutional ethical guidelines. Informed consent was obtained in accordance with European Union guidelines for biomedical research. The study was approved by the Human Ethics Committee of the Hospital Universitari Germans Trias i Pujol. Tumor tissues from HB patients obtained at the time of surgical resection were used to generate organoids and plated in 50 μL of BME (Basement Membrane Extract, Type 2, Pathclear, Bio‐Techne, Minneapolis, MN, USA), as described previously [23]. Organoids were expanded once a week through mechanical and enzymatic disruption and plated in a new BME drop. After mechanical disruption, organoids were resuspended in 10 μL of BME and dispensed into white, clear flat‐bottom 96‐well plates (#3610, Costar, Corning, NY, USA). Organoids were treated with media supplemented with either WNTinib (5 μm), cisplatin (3 μm; #232120; Sigma‐Aldrich, St. Louis, MO, USA ) or vehicle for 7 days. Cell viability was assessed using CellTiter‐Glo (Promega, Madison, WI, USA) according to the manufacturer's instructions. Procedures were approved by the Ethics Committee of the Hospital Clinic of Barcelona, Spain (protocol number HCB‐2019‐0041), and informed consent was obtained from all patients (or their legal representative).

### 2.6 Genomic sequencing

The *CTNNB1* status for the five HB cell lines derived from the PDX murine models from the University of Texas, Health Science Center at San Antonio (COG#891173, 0543–000, 0498‐FT0921, 1925, 0648‐000) was determined using Sanger Sequencing (Table S1). To this end, genomic DNA was extracted from the six cell lines using the DNeasy Blood & Tissue Kit (QIAGEN, Germantown, MD, USA). The target genomic region was amplified by PCR using forward 5′‐(GTGAGTAACTGTTAGGTGGTTCC)‐3′ and reverse 5′‐(GTCAGTTCAGGGATTGCACG)‐3′ primers (Sigma‐Aldrich, St. Louis, MO, USA). PCR was performed using Herculase II Fusion DNA polymerase (Agilent, Santa Clara, CA, USA) according to the manufacturer's instructions and 1 μg of genomic DNA as a template. PCR conditions were: 95 °C for 2 min, 95 °C for 30 s,  60.7 °C for 30 s, 72 °C for 1 min × 34 cycles, 72 °C for 8 min. The PCR product was column purified (QIAGEN, Germantown, MD, USA) and submitted for Sanger sequencing (Psomagen, New York, NY, USA).

### 2.7 Statistical methods

Statistical analyses and graphical presentations were performed using R (version ≥4.0.2). Shapiro–Wilk tests were used to assess normality. Parametric tests (Student's *t*‐test or ANOVA) were applied to normally distributed data, while non‐parametric tests (Wilcoxon or Kruskal–Wallis) were used for non‐normally distributed data. Binary counts were assessed through Fisher's exact test. Differences between treatment groups were assessed with Kaplan–Meier estimates and a log‐rank test. Overall, *P*‐value < 0.05 or FDR < 0.05 for multiple comparisons was considered significant.

### 2.8 Animal studies

All animals were healthy and acclimated to the animal facility prior to the start of the experiments. Animals were housed in temperature‐ and humidity‐controlled rooms on a 12‐h light/dark cycle, with free access to standard rodent chow and water *ad libitum*, and were group‐housed in cages with appropriate bedding and environmental enrichment. Studies were performed in compliance with guidelines for the use of animals established by the institution's ethical committee and the “Guide for the Care and Use of Laboratory Animals” and were approved by the Icahn School of Medicine at Mount Sinai Institutional Animal Care and Use Committee (protocol number IACUC‐2018‐0013). Animals were monitored daily for signs of distress or illness, and any moribund animals were humanely euthanized following the Guidelines for Humane Endpoints for Animals Used in Biomedical Research and the Declaration of Helsinki.

## 3 Results

### 3.1 WNTinib elicits antitumoral effects and extends survival in PDX‐derived HB murine models

**Fig. 1:**
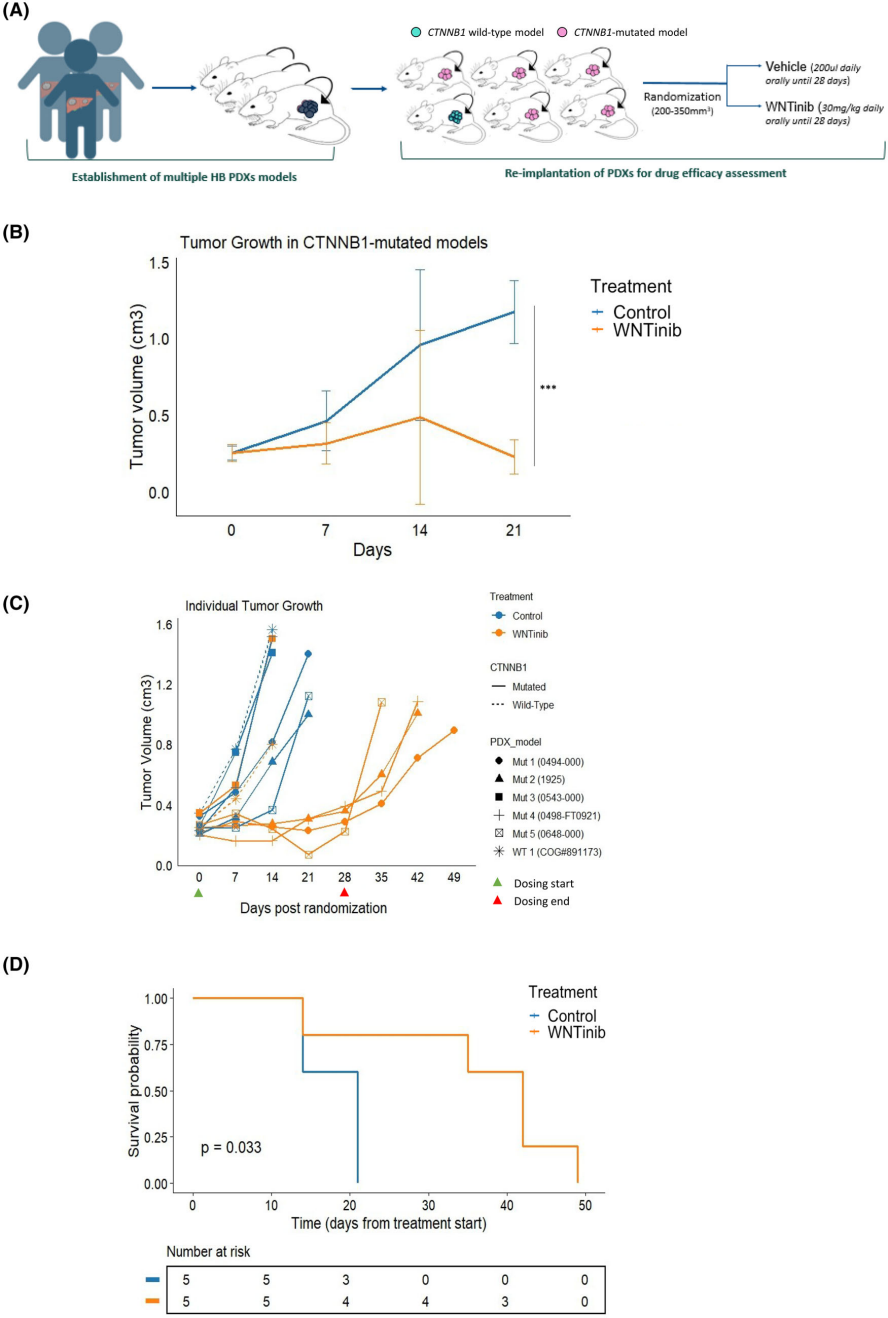
Efficacy of WNTinib in PDX models of HB. (A) Experimental design and methodology of the murine PDX model. (B) Tumor growth in *CTNNB1*‐mutated PDX models treated with WNTinib (*n* = 5) or control (*n* = 5). Statistics: Wilcoxon rank sum test; ***P < 0.001. Error bars indicate standard error of mean. (C) Individual tumor growth curves for each animal categorized by *CTNNB1* mutation status (solid line – *CTNNB1* mutated, dashed line – *CTNNB1* wild type); PDX model (all six PDX models represented with different symbols); and treatment arm (WNTinib depicted in orange, control—in blue). (D) Kaplan–Meier overall survival analysis in the *CTNNB1*‐mutated PDX models treated with WNTinib (*n* = 5) or control (*n* = 5). Statistical significance was tested using a log‐rank test.

The antitumor potential of WNTinib inhibition was tested in six different HB PDX murine models generated through the implantation of six distinct patient‐derived xenografts (Fig. 1A). Among these, five of the implanted tumors harbored *CTNNB1* mutations, while one model exhibited *CTNNB1* wild‐type (WT) status (Table S1). Mice with HB PDX tumors were randomly allocated to receive either WNTinib or vehicle. No differences in tumor volumes were observed between the two treatment arms at the point of randomization (Fig. S1A). In this context, WNTinib significantly reduced tumor growth in *CTNNB1*‐mutated models (*P* = 0.0005; day 21) (Fig. 1B). In addition, WNTinib efficiently delayed tumor progression in 80% (*n* = 4/5) of *CTNNB1‐mutated* models until the end of the treatment regimen (day 28), whereas this effect was not observed in the *CTNNB1* WT model (Fig. 1C). Moreover, *CTNNB1* HB models demonstrated significantly increased survival, with a median survival in the WNTinib‐treated group twice as long as the control group (42 vs 21 days, log‐rank *P* = 0.033) (Fig. 1D).

### 3.2 WNTinib treatment exhibits antitumor activity and enhances survival in two independent HB murine models

**Fig. 2:**
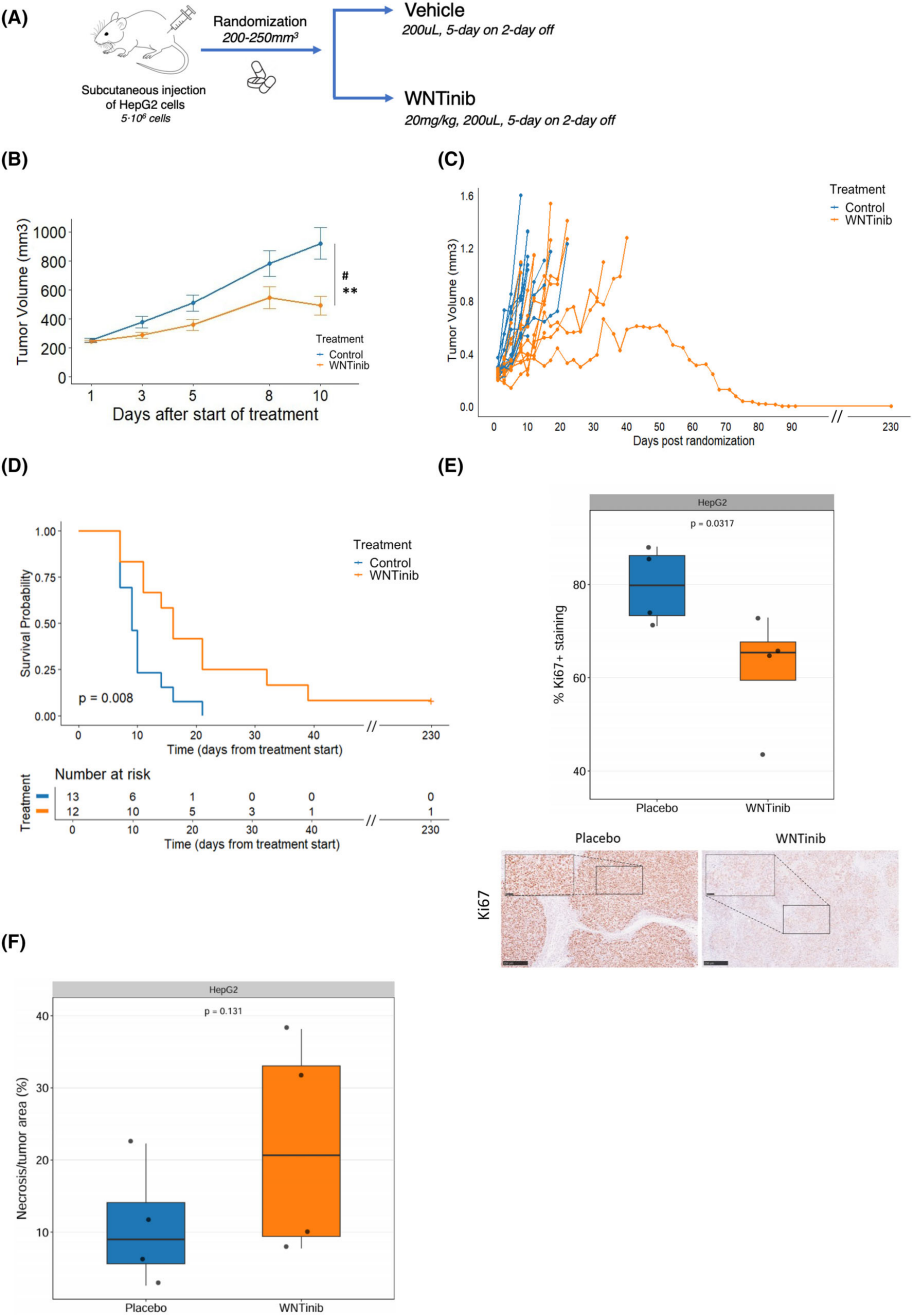
Efficacy of WNTinib in the HepG2 xenograft model. (A) Schematic summary of the experimental design of the HepG2 xenograft model. (B) Tumor growth plot. Statistics: Wilcoxon rank sum test; #*P* < 0.05 (Day 8); **P < 0.001 (Day 10). Error bars indicate the standard error of the mean. (C) Spaghetti plot showing tumor growth for each animal, with WNTinib‐treated (orange) mice and control‐treated (blue) mice. (D) Kaplan–Meier survival analysis in animals treated with WNTinib (*n* = 13) or control (*n* = 12). Statistical significance was tested using a log‐rank test. (E) Quantification of Ki67 positive immunohistochemistry staining in HepG2‐derived HB tumors (*n* = 4/arm) with representative images. Whiskers represent maximum (75th percentile + 1.5 × interquartile range) and minimum (25th percentile – 1.5 × interquartile range) values. Statistics: One‐way Student's t‐test. Scale bar, 250 μm. (F) Quantification of necrosis normalized to the total tumor area. Whiskers represent maximum (75th percentile + 1.5 × interquartile range) and minimum (25th percentile – 1.5 × interquartile range) values. Statistics: One‐way Student's t‐test.

**Fig. 3:**
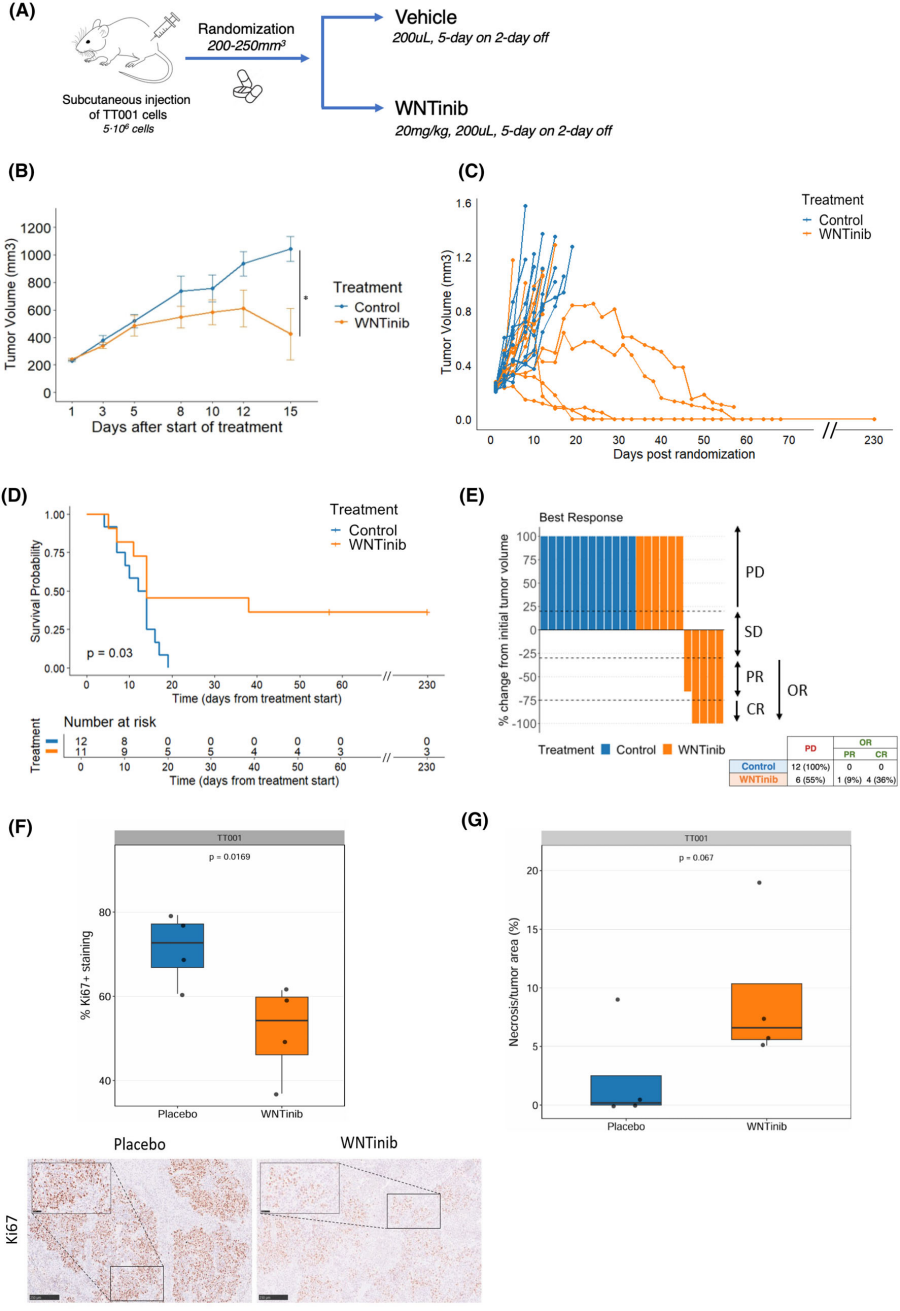
Efficacy of WNTinib in the TT001 xenograft model. (A) Schematic summary of the experimental design of the TT001 xenograft model. (B) Tumor growth plot. Statistics: Wilcoxon rank sum test; *P < 0.05 (Day 15). (C) Spaghetti plot showing tumor growth for each animal, with WNTinib‐treated (orange) mice and control‐treated (blue) mice. (D) Kaplan–Meier survival analysis in animals treated with WNTinib (*n* = 12) or control (*n* = 11). Statistical significance was tested using a log‐rank test. (E) Waterfall plot of tumor response at best response according to mRECIST criteria. The Y‐axis is capped at 100%. CR, complete response; PR, partial response; OR, objective response; SD, stable disease; PD, progressive disease. (F) Quantification of Ki67 positive immunohistochemistry staining in TT001‐derived HB tumors (*n* = 4/arm) with representative images. Whiskers represent maximum (75th percentile + 1.5 × interquartile range) and minimum (25th percentile – 1.5 × interquartile range) values. Statistics: One‐way Student's t‐test. Scale bar, 250 μm. (G) Quantification of necrosis normalized to the total tumor area. Whiskers represent maximum (75th percentile + 1.5 × interquartile range) and minimum (25th percentile – 1.5 × interquartile range) values. Statistics: One‐way Student's t‐test.

To further assess the antitumor efficacy of WNTinib in HB, we generated two *CTNNB1* mutated xenograft murine HB models (Figs 2A, 3A), one with the immortalized human HepG2 cell line (*n* = 25) and one with the TT001 cell line (*n* = 23) established from HB PDX tissue. Mice bearing tumors were randomized to either receive WNTinib (HepG2 *n* = 12; TT001 *n* = 11) or vehicle (HepG2 *n* = 13; TT001 *n* = 12). Median tumor volume at randomization was equal in all treatment arms and no significant differences in toxicity signs were observed (Fig. S1B,C; Fig. S2B,C).

In the HepG2 model, WNTinib displayed a significant delay of tumor growth from the initiation of the treatment compared to the control arm (*P* = 0.03 and *P* = 0.002 on days 8 and 10 of the treatment, respectively) (Fig. 2B). Interestingly, response to WNTinib was bimodal, with some animals experiencing fast progressions as the control mice and some others delaying tumor growth. WNTinib treatment showed a nonsignificant trend to reduce the number of fast progressing (20 days) animals and reaching survival endpoint 7 out of 12 (58%) WNTinib‐treated vs 12 out of 13 (92%) in the control group (*P* = 0.073) (Fig. 2C). Consistently, mice treated with WNTinib exhibited a significantly improved survival compared to control (log‐rank *P* = 0.008), with the median survival for the WNTinib‐treated group being 16 days vs 9 days for the control group (Fig. 2D), and one long lasting survivor (>230 days) in the treatment group.

In the TT001 model, WNTinib also significantly delayed tumor growth compared to the control group (*P* = 0.02, day 15) (Fig. 3B). In addition, 55% (*n* = 6/11) of the WNTinib‐treated mice reached the survival endpoint (day 20) in contrast to 100% (*n* = 12/12) of the control animals (*P* = 0.014) (Fig. 3C). In line, WNTinib‐treated mice demonstrated significantly increased survival (log‐rank *P* = 0.03), including 3 long lasting (>230 days) survivors (Fig. 3D). Notably, 45% (*n* = 5/11) of WNTinib‐treated animals achieved an objective response (OR), as opposed to the control group where none of the animals achieved an OR (*P* = 0.014 vs control), with 80% (*n* = 4/5) of the animals with an OR being complete responders (CR) according to mRECIST criteria (*P* = 0.037 vs control) (Fig. 3D). Consistently, immunohistochemical staining for Ki67 revealed a significant reduction in proliferative activity in WNTinib‐treated tumors compared with controls in both HepG2 (*P* = 0.032) and TT001 (*P* = 0.017) models (Figs 2E, 3F). In parallel, histological evaluation revealed a trend toward increased necrotic areas in WNTinib‐treated tumors, supporting the antiproliferative effect of WNTinib *in vivo*, in line with that previously reported in HCC (Figs 2F, 3G) [18].

Overall, WNTinib demonstrated antitumoral efficacy in three independent HB experimental models with a tolerable safety profile and no significant signs of animal welfare deterioration.

### 3.3 WNTinib elicits antitumoral activity in *CTNNB1* ‐mutated HB patient‐derived organoids

**Fig. 4:**
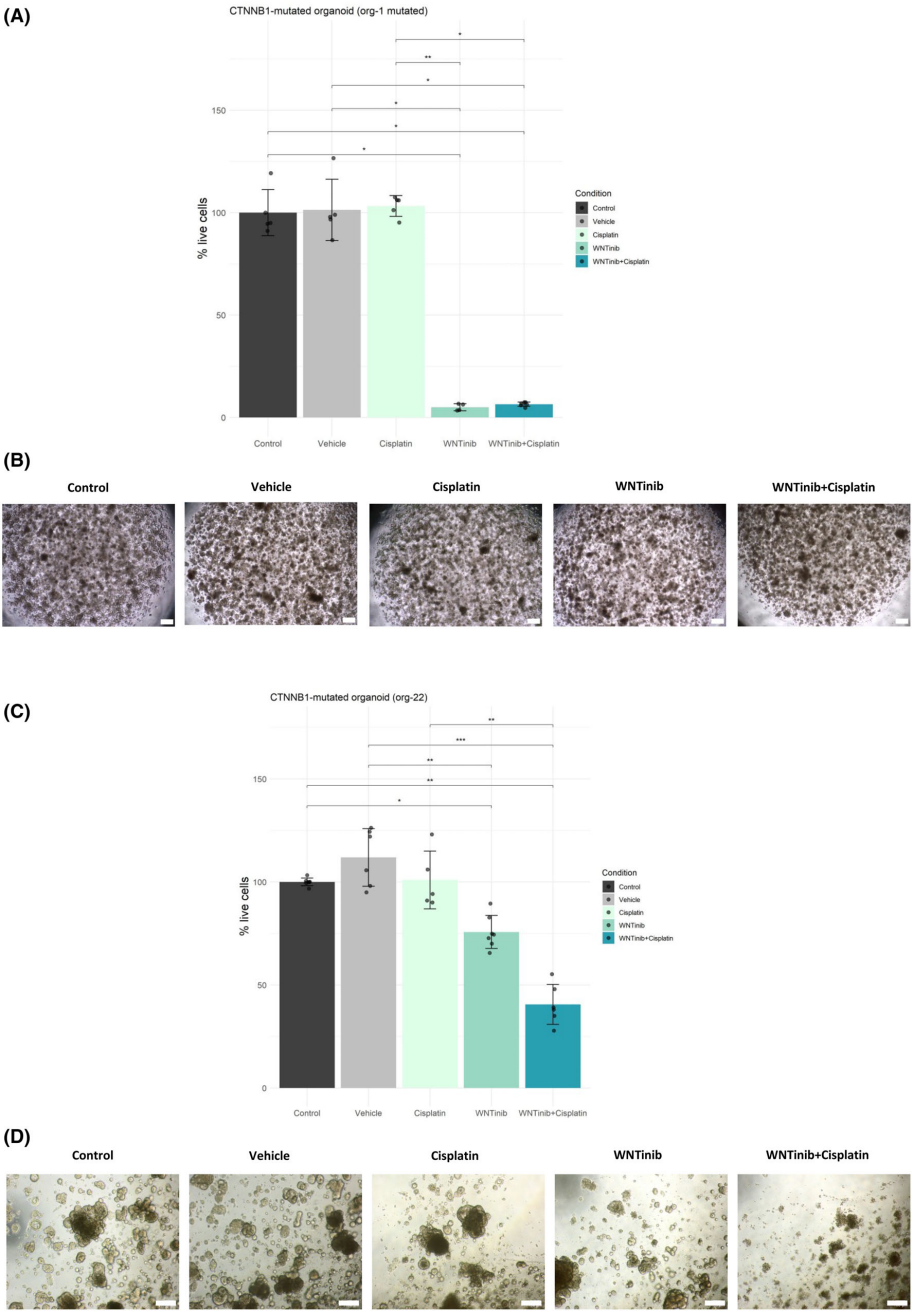
Efficacy of WNTinib in patient‐derived HB organoids. Cell viability in two models of *CTNNB1‐mutated* HB tumor organoids treated with cisplatin (3 μm), WNTinib (5 μm) and their combination. (A) Viability of org‐1 following 7‐day treatment. Error bars indicate standard deviation. (B) Representative images of org‐1. Scale bar = 200 μm. (C) Viability of org‐22 following 7‐day treatment. Error bars indicate standard deviation. (D) Representative images of org‐22. Scale bar = 200 μm. Images were captured at 4× magnification. Statistics: Kruskal–Wallis test followed by the Dunn test adjusted by BH; *P < 0.05; **P < 0.01; ***P < 0.001.

WNTinib's efficacy was further validated in two established patient‐derived HB organoid models that harbor *CTNNB1* mutations (Table S2). Organoids were treated with WNTinib, cisplatin, a combination of both, or vehicle; and organoid viability was assessed. A significant viability reduction was observed in both organoid models following treatment with WNTinib, and both resulted resistant to the standard of care, cisplatin. In particular, org‐1 showed a profound response to WNTinib, with a 95% reduction in viability compared to 100% viability in vehicle or control (*P* < 0.05) (Fig. 4A,B). Org‐22 responses to WNTinib were more modest, yet significant compared to vehicle (*P* < 0.01, in org‐22), with only ~30% reduction in viable cells (Fig. 4C,D). The combination of WNTinib with cisplatin further enhanced the antitumor effect of WNTinib, resulting in a 93.5% reduction in org‐1 viability (*P* < 0.05 vs vehicle; *P* < 0.05 vs cisplatin) and a 59.5% reduction in org‐22 viability (*P* < 0.001 vs vehicle; *P* < 0.01 vs cisplatin).

These data obtained using HB organoids are consistent with our findings observed in HB murine models, collectively demonstrating that WNTinib effectively impairs tumor growth across multiple *CTNNB1*‐mutated HB models, and supports the potential of WNTinib for further evaluation in clinical trials.

## 4 Discussion

Several studies have defined the molecular characteristics of HB and profiled its main genetic and epigenetic alterations [9, 10, 11, 24, 25, 26], however, precision oncology has not yet been introduced into its clinical management. Here, we demonstrated that WNTinib exhibits antitumoral efficacy, extending survival specifically in *CTNNB1‐*mutated PDX models. These findings were further validated in additional *CTNNB1*‐mutated murine models using two different HB cell lines. Finally, we confirmed these *in vivo* results using patient‐derived HB organoids harboring the *CTNNB1* mutation, where WNTinib proved effective as a single agent and in combination with cisplatin.

Our study is relevant as it provides a potential alternative path for HB patients. Adjuvant and neoadjuvant cisplatin‐based chemotherapy has been shown to improve clinical outcomes following surgical resection in HB patients [27]. However, the 3‐year recurrence‐free survival rate for patients with advanced‐stage disease remains only 34% [4]. In addition, these chemotherapy regimens are associated with severe and often lifelong toxicities, including ototoxicity [5, 6], and cardiomyopathies [6]. We and others reported that *CTNNB1* is the most recurrent molecular alteration in HB [9, 10], with up to 70–90% of patients presenting with alterations in the WNT/β‐catenin pathway. Therefore, inhibitors of this pathway are largely awaited. Several Wnt pathway inhibitors have been explored in different cancer types [28], including Wnt ligands or receptors inhibitors [29, 30, 31], enhancers of the β‐catenin destruction complex [32, 33], or repressors of the β‐catenin transcriptional activity [34]. However, none of them reached clinical practice, mainly due to a lack of efficacy or high toxicity in clinical trials [35]. WNTinib is a WNT/β‐catenin‐pathway kinase inhibitor that previously demonstrated antitumor activity and a safety profile in *CTNNB1*‐mutated preclinical models of HCC [18].

Our data derived from the HB patient‐derived organoids suggest that WNTinib has a higher antitumoral efficacy compared to cisplatin, the standard perioperative treatment in HB management (~90 to ~30% viability reduction, depending on the organoid model). These results are consistent with results in *CTNNB1*‐mutant HCC organoids previously reported [18]. Furthermore, WNTinib combined with cisplatin was able to overcome the lack of antitumoral efficacy of cisplatin alone in these two cisplatin‐resistant models (~90 to ~60% viability reduction for the cisplatin‐WNTinib combination, depending on the organoid model), and could represent a promising therapeutic strategy for the ~20% of patients who are refractory to these standard chemotherapy regimens [1, 10]. Further preclinical studies on xenografts or orthotopic *in vivo* models would be required to validate the results obtained in our organoid models.

In addition, our *in vivo* data demonstrating WNTinib efficacy in murine models of *CTNNB1*‐mutant HB tumors parallels previously reported data in HCC models harboring β‐catenin mutations. Notably, in the TT001 model [22], WNTinib achieved a complete response in over one‐third of animals, highlighting the potential of WNTinib to reach durable tumor regression in patients with *CTNNB1*‐driven HB. Further experiments are warranted to investigate which mechanisms contribute to the differential treatment responses among *CTNNB1*‐mutated models.

## 5 Conclusions

Our study provides preclinical evidence that *CTNNB1*‐mutated HB—which accounts for up to 90% of pediatric cases—may be actioned with personalized therapy via Wnt signaling inhibition. Also, this research study provides the rationale for exploring WNTinib as a therapy to increase response rates of HB patients with a safer profile. Finally, the combination of WNTinib with the standard of care warrants further investigation, to provide additional mechanistic insights and translational relevance. Such studies may also enable the reduction of cisplatin dose and mitigate chemotherapy‐associated side effects in patients with *CTNNB1*‐mutant HB.

## Conflict of interest

JML is receiving research support from Genentech and Roche, consulting fees from Eisai Inc., Merck, Roche, Genentech, AstraZeneca, Bayer Pharmaceuticals, Abbvie, Sanofi, Moderna, Glycotest, Exelixis and Boehringer Ingelheim and participates in the Data Safety Monitoring Board for Industry with Bristol Myers Squibb.

## Author contributions

EG and JML designed and supervised the study. UB, JHP, JAF, EFM, VdP, and LZ performed experiments/analysis. UB, JHP, JAF, AGO, AR, EFM, CM, VdP, PH, LZ, AM, IK, ST, CA, PSB, EG, RP, and JML provided scientific input. The manuscript was written by UB, JHP, JAF, AGO, and RP under the supervision of JML. All authors were involved in the critical revision of the manuscript.

## Supporting information


**Fig. S1.** Tumor volumes at randomization across HB PDX, HepG2, and TT001 models. (A) Tumor volumes of HB PDX models at randomization point per treatment. (B) Tumor volumes of HepG2 model at randomization point per treatment. (C) Tumor volumes of TT001 model at randomization point per treatment. Statistics: Wilcoxon rank sum test. n.s., nonsignificant.
**Fig. S2.** Body weight progression during treatment across PDX, HepG2, and TT001 models. (A) Spider plot showing the body weight progression of individual animal for each treatment in the PDX model. The survival endpoint (1000 mm^3^ tumor volume) led to early sacrifice in the control arm before treatment completion. (B) Body weight progression is shown as a percentage and normalized to the weight at the point of randomization in HepG2 model. Error bars indicate standard error of mean. (C) Body weight progression is shown as a percentage and normalized to the weight at the point of randomization in the TT001 model. Error bars indicate standard error of mean. Statistics: Wilcoxon rank sum test. n.s., nonsignificant.

**Table S1.** *CTNNB1* mutation status and histology type of the PDX.
**Table S2.** Clinical and histological assessment of tumor‐derived org‐1 and org‐22.

## Data Availability

The data that support the findings of this study are available upon request to the corresponding author. The data are not publicly available due to privacy or ethical restrictions.
