# Supplementary figures and images for: Effective therapeutic targeting of CTNNB1‐mutant hepatoblastoma with WNTinib
Source: Mol Oncol. 2025 Dec 8;20(4):920–32. doi: 10.1002/1878-0261.70168 (PMC13060634; doi:10.1002/1878-0261.70168)

# Supplementary Figure 1.

A

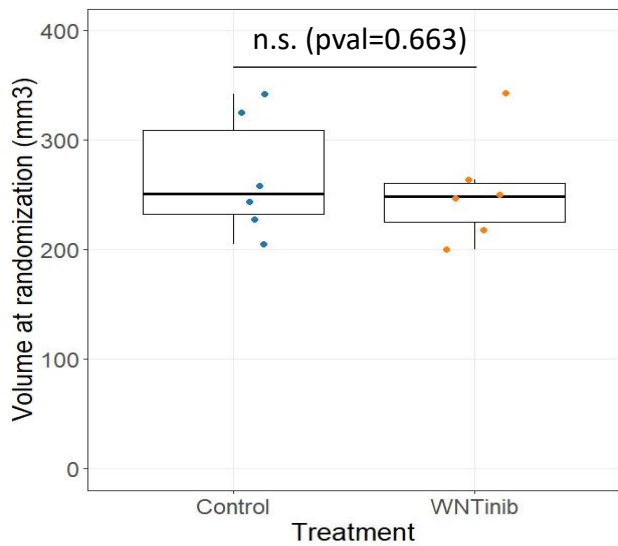

B

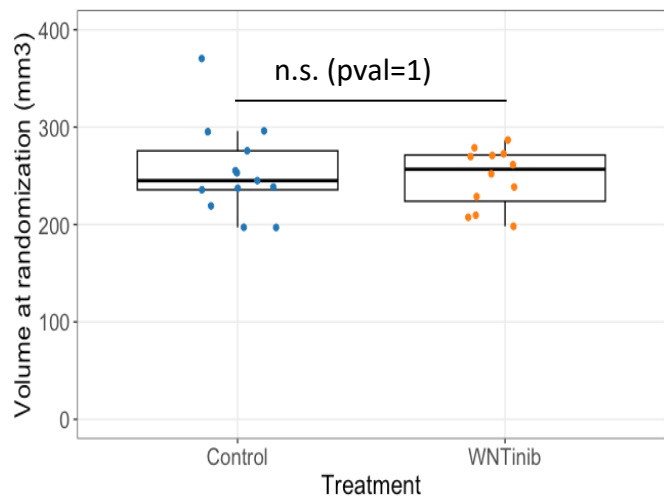

C

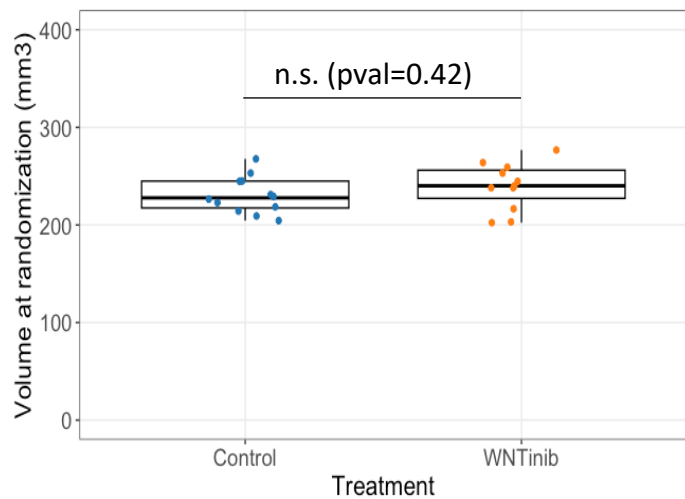

# Supplementary Figure 2.

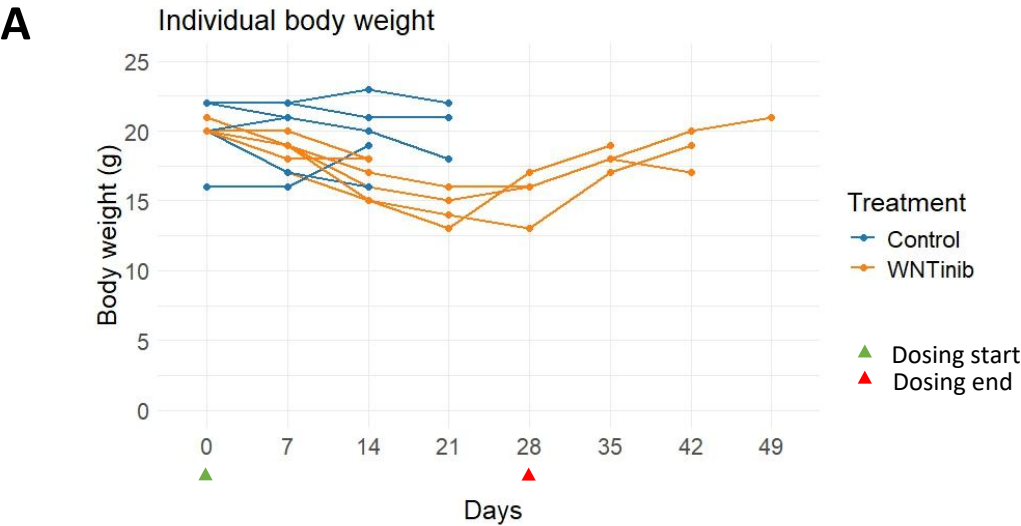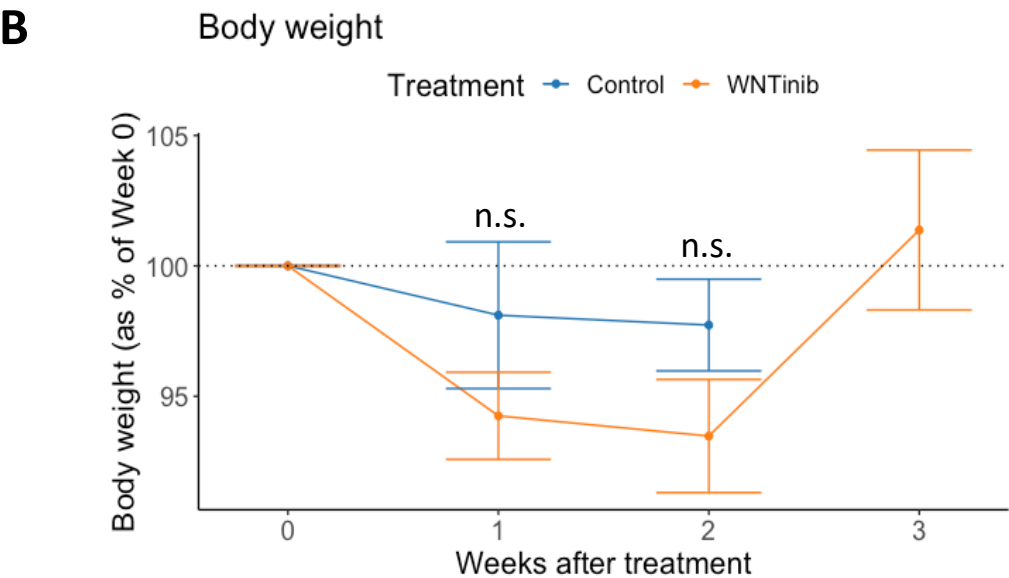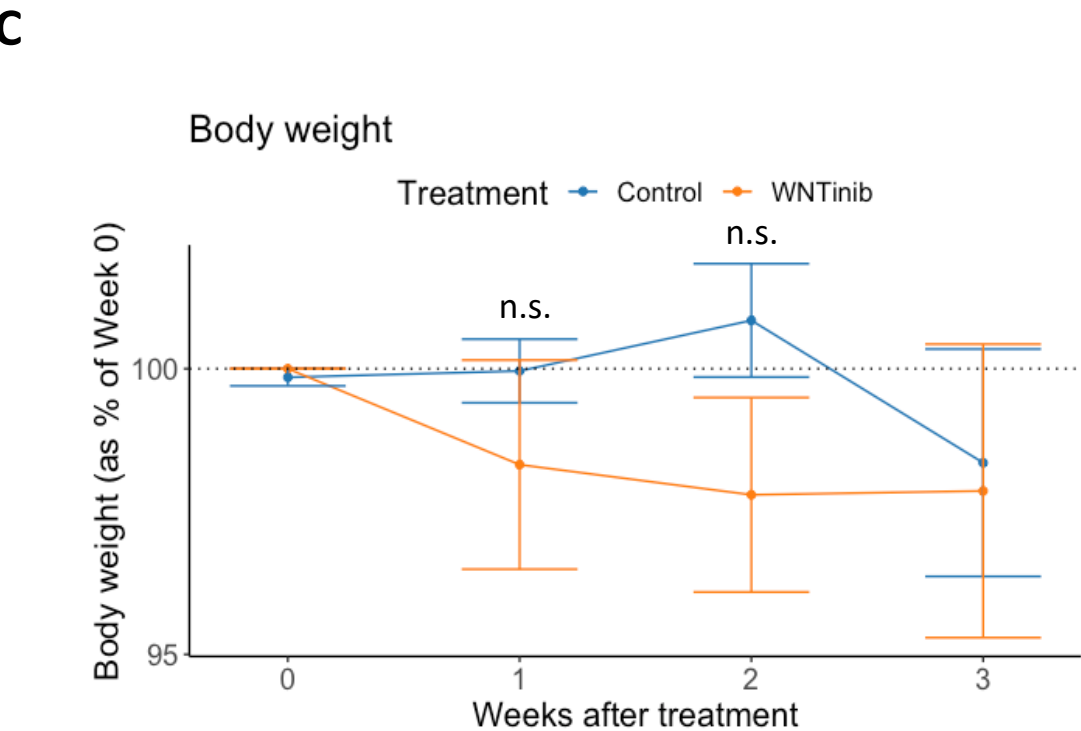

Supplement: Supplementary file 1 — Fig. S1. Tumor volumes at randomization across HB PDX, HepG2, and TT001 models. (A) Tumor volumes of HB PDX models at randomization point per treatment. (B) Tumor volumes of HepG2 model at randomization point per treatment. (C) Tumor volumes of TT001 model at randomization point per treatment. Statistics: Wilcoxon rank sum test. n.s., nonsignificant. Fig. S2. Body weight progression during treatment across PDX, HepG2, and TT001 models. (A) Spider plot showing the body weight progression of individual animal for each treatment in the PDX model. The survival endpoint (1000 mm3 tumor volume) led to early sacrifice in the control arm before treatment completion. (B) Body weight progression is shown as a percentage and normalized to the weight at the point of randomization in HepG2 model. Error bars indicate standard error of mean. (C) Body weight progression is shown as a percentage and normalized to the weight at the point of randomization in the TT001 model. Error bars indicate standard error of mean. Statistics: Wilcoxon rank sum test. n.s., nonsignificant. [file MOL2-20-920-s001.pdf]
