# Supplementary material for: Effective therapeutic targeting of CTNNB1‐mutant hepatoblastoma with WNTinib
Source: Mol Oncol. 2025 Dec 8;20(4):920–32. doi: 10.1002/1878-0261.70168 (PMC13060634; doi:10.1002/1878-0261.70168)
Supplement: Supplementary file 2 — Table S1. CTNNB1 mutation status and histology type of the PDX. Table S2. Clinical and histological assessment of tumor‐derived org‐1 and org‐22. [file MOL2-20-920-s002.pdf]

Supplementary Table 1. *CTNNB1* mutation status and histology type of the PDX

PDX ID	PDX reference	*CTNNB1* mutation	Histology type
Mut1	0494-000	G34E, missense	Predominantly embryonal
Mut2	1925	Exon 3 deletion	Embryonal
Mut3	0543-000	G34E, missense	Predominantly embryonal
Mut4	0498-FT0921	Exon 3 deletion	Predominantly embryonal
Mut5	0648-000	S33Y, oncogenic	Mixed: fetal, embryonal
wt	Cog#891173	WT	Predominantly embryonal

Supplementary Table 2. Clinical and histological assessment of tumor-derived org-1 and org-22

	Diagnosis	*CTNNB1* mutation	Histology	Main epithelial component
**org-1**	Hepatoblastoma	S33C, oncogenic	Epithelial	Macrotrabecular
**org-22**	Hepatoblastoma	Exon 3 deletion and Q28H missense	Mixed	Embryonal & Pleomorphic & Fetal Crowded
